# Preserved perception-action integration in adolescents after a COVID-19 infection

**DOI:** 10.1038/s41598-023-40534-6

**Published:** 2023-08-16

**Authors:** Katharina Graf, Alena Gustke, Mariella Mösle, Jakob Armann, Josephine Schneider, Leonie Schumm, Veit Roessner, Christian Beste, Annet Bluschke

**Affiliations:** 1 Cognitive Neurophysiology, Department of Child and Adolescent Psychiatry, Faculty of Medicine, TU Dresden, Schubertstrasse 42, 01309 Dresden, Germany; 2 University Neuropsychology Center (UNC), Faculty of Medicine, TU Dresden, Dresden, Germany; 3 Department of Paediatrics, University Hospital Carl Gustav Carus, Technische Universität Dresden, Dresden, Germany

**Keywords:** Human behaviour, Viral infection, Cognitive neuroscience, Cognitive control

## Abstract

Evidence is accumulating that the Coronavirus disease (COVID-19) can bring forth deficits in executive functioning via alterations in the dopaminergic system. Importantly, dopaminergic pathways have been shown to modulate how actions and perceptions are integrated within the brain. Such alterations in event file binding could thus underlie the cognitive deficits developing after a COVID-19 infection. We examined action-perception integration in a group of young people (11–19 years of age) that had been infected with COVID-19 before study participation (n = 34) and compared them to a group of uninfected healthy controls (n = 29) on the behavioral (i.e., task accuracy, reaction time) and neurophysiological (EEG) level using an established event file binding paradigm. Groups did not differ from each other regarding demographic variables or in reporting psychiatric symptoms. Overall, multiple lines of evidence (behavioral and neurophysiological) suggest that action-perception integration is preserved in adolescents who suffered from COVID-19 prior to study participation. Event file binding processes were intact in both groups on all levels. While cognitive impairments can occur following a COVID-19 infection, the study demonstrates that action-perception integration as one of the basic building blocks of cognition seems to be largely unaffected in adolescents with a rather mild course of the disease.

## Introduction

The global impact of the Coronavirus Disease 2019 (COVID-19), a syndrome caused by the Severe Acute Respiratory Syndrome Coronavirus 2 (SARS-CoV-2), on people’s physical and mental health is becoming increasingly evident. Next to reported acute, but also persistent, physical^1,2^ and psychiatric^3,4^ symptoms, evidence is accumulating that the disease might also bring forth cognitive symptoms^5,6^.

In neuropsychological studies and interviews, especially deficits in executive functioning have been reported^3,7–10^. More specifically, impairments in inhibition, sustained and selective attention, set-shifting, and abstraction have been described^3^. Notably, research until now focused mainly on severe cases even though research shows that cognitive dysfunction can occur after a COVID-19 infection independent of disease severity^11–14^. Further, most studies so far concern the impact of COVID-19 on adult populations, with the impact on cognitive processes in children still being largely elusive^15^. Additionally, research until now has mainly focussed on the purely clinical/behavioural level, with only very little being known about the underlying mechanisms and neurophysiological processes.

Next to more general explanations^16^, a more specific proposed explanatory approach concerning the occurrence of cognitive sequelae focuses on dopamine^17,18^. The angiotensin-converting enzyme 2 (ACE-2) receptor allows COVID-19 to access human cells in the central and peripheral nervous system. By doing so, the virus also enters and infects brain regions that are important for the dopamine synthesis in the ventral tegmental area, the substantia nigra and the hypothalamus^19,20^. This mechanism possibly triggers degeneration of dopaminergic neurons leading to an altered dopamine concentration in the brain^17^. Importantly, the dopaminergic system is closely associated with functions in various cognitive domains^21^. Specifically, perception–action integration as one of the basic building blocks of cognition^22^ is of interest here due to its close relationship to dopaminergic neurotransmission^23–27^. The integration of perception and action is fundamental for any form of goal-directed behaviour^28,29^ and thus also for daily life abilities^30^. The theory of event coding (TEC^22,31^) offers a well-established framework for analysing the integration of perception and action. According to TEC, perceptual stimuli and actions share a so-called ‘common coding mechanism’^32^. This results in the establishment of so-called ‘event files’ comprising response- and stimulus-features as well as the links and associations between them. Aside from the behavioural level (i.e., reaction times and accuracy), correlates of event-file binding on the neurophysiological level can also be examined, allowing a much closer consideration of the underlying processes probably affected by SARS-COV-2 infection. Importantly it has been shown that, presumably based on the reduction of intra-individual variability, event file binding functions more efficiently in adults compared to children^33,34^.

In the current study, we ask whether and how a SARS-COV-2 infection in childhood and adolescence affects perception–action integration. Perception–action integration was investigated utilizing an adapted version of the stimulus-response (S-R) task^35^. In order to elucidate the underlying neurophysiological mechanisms, we employed EEG techniques in addition to analysing behavioural data. To account for intra-individual variability and be able to differentiate between different coding levels in the EEG signal during event file processing^36,37^, we applied residue iteration decomposition (RIDE) to the EEG data^38,39^. RIDE decomposes the EEG signal into functionally separate “clusters” of activity^37^, with the S-cluster referring to stimulus-related processes like perception and attention, the R-cluster representing response-related processes like motor planning and execution, and the C-cluster reflecting intermediate processes connecting the S- and R-cluster^38–40^. The C-cluster in particular appears to reflect stimulus-response association mechanisms^40,41^, making it specifically appealing for studying event file binding in COVID-19. In fact, several line of evidence provide direct evidence that the C-cluster specifically reflects event file binding dynamics^42–44^. Considering that SARS-COV-2 has been reported to lead to cognitive dysfunctions via alterations in the dopaminergic system^7,17^ and the configuration of event files has also been shown to be modulated by dopaminergic pathways^23–27^, we hypothesized that a reduced level of dopamine concentration within the brain caused by SARS-CoV-2 results in impaired action-perception integration processes in children and adolescents following a COVID-19 infection. More specifically, younger people who were infected prior to the study are hypothesized to show reduced event file binding processes on the behavioural and neurophysiological level (reduced binding effects on the P3 window in the C-cluster) compared to healthy controls.

## Results

### Behavioural data

#### Task accuracy

**Figure 1:**
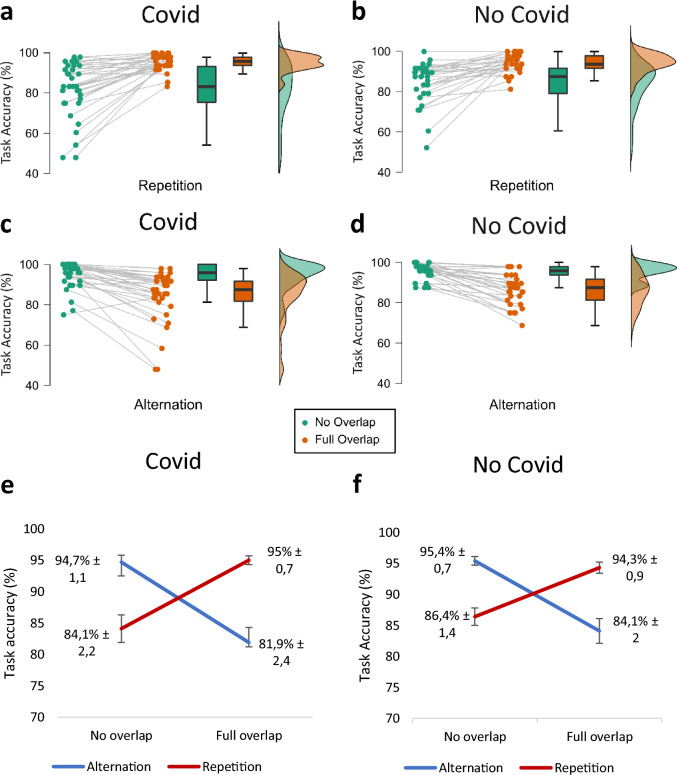
Raincloud Plots and Line Graph of the Task Effect in the Covid (**a**, **c**, **e**) and No Covid (**b**, **d**, **f**) Group for Task Accuracy*.* Task accuracy displays the percentage of correct responses (m ± SE).

It was shown that while there was a significant main effect of feature overlap (*F*(1,61) = 4.815, *p* = 0.032, *η*_*p*_^2^ = 0.073, *BF*_01_ =  < 0.001), neither a significant main effect of response type nor a significant main effect of group was present (all *F*’s < 2.051, all *p*’s > 0.157, all *η*_*p*_^2^ < 0.033). All participants responded more accurately when there was no feature overlap (90.1% ± 0.9) compared to when there was a full feature overlap (88.8% ± 0.9). Furthermore, there was a significant interaction effect between response type and feature overlap (*F*(1,61) = 91.135,* p* =  < 0.001, *η*_*p*_^2^ = 0.599, *BF*_01_ =  < 0.001). Therefore, these results confirm that a binding effect has been established by the task, and thus validate the experimental approach in the current sample. Please refer to Fig. 1 for more detailed information about this task effect in the Covid and No Covid group. Importantly, there was no significant interaction effect between group × feature overlap × response type on the behavioural level (*F*(1,61) = 1.017, *p* = 0.317, *η*_*p*_^2^ = 0.016, *BF*_01_ = 10.997) and the Bayesian analysis revealed strong evidence for the lack of effects. Thus, there is robust evidence that, on the level of task accuracy, the process of event file binding does not differ between healthy participants and participants that have been infected with Covid-19 prior to the study.

#### RTs

**Figure 2:**
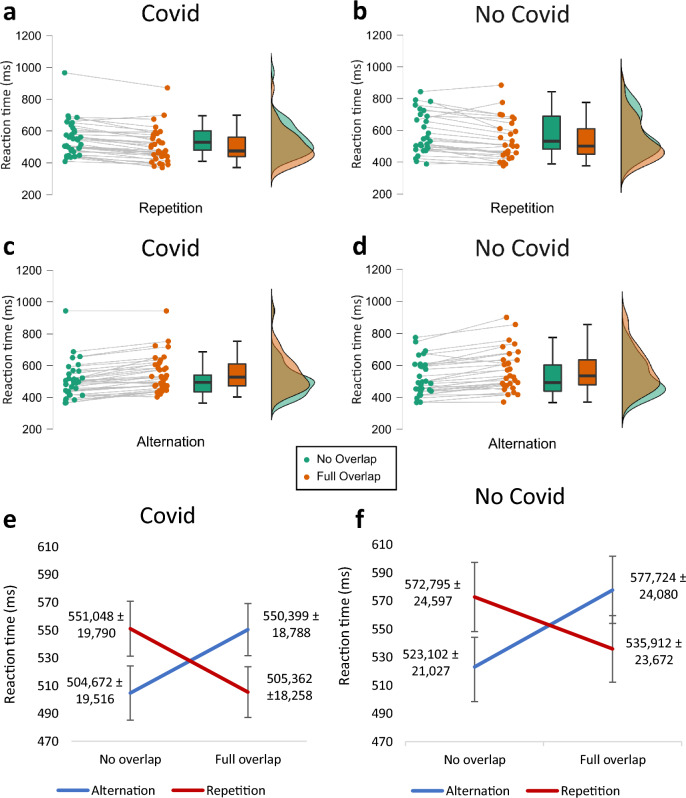
Raincloud Plots and Line Graph of the Task Effect in the Covid (**a**, **c**, **e**) and No Covid (**b**, **d**, **f**) Group for Reaction time*.* Reaction time was measured in milliseconds (ms; m ± SE).

For the reaction times, there are no significant main effects for either response type, feature overlap, or group (all *F*’s < 1.539, all *p*’s > 0.219, all *η*_*p*_^2^ < 0.025). However, similar to the accuracy measure, the results suggest that there was a significant interaction effect between response type and feature overlap, *F*(1,61) = 175.490, *p* < 0.001, *η*_*p*_^2^ = 0.742, *BF*_01_ =  < 0.001. Please refer to Fig. 2 for a complete overview of these effects. Importantly, the analysis concerning the reaction times also revealed no significant interaction effect between group x response type x feature overlap (*F*(1,61) =  < 0.001, *p* = 0.995, *η*_*p*_^2^ < 0.001, *BF*_01_ = 8.783). Bayesian statistics revealed substantial evidence for the null hypothesis for this interaction effect.

#### Regression analyses

Furthermore, a backward stepwise linear regression was calculated for each condition (no overlap—alternation, no overlap—repetition, full overlap—alternation, full overlap—repetition) to explore the influence of potential predictors (vaccination status, serostatus, titre value, IQ) on participant’s task performance on a behavioural level (task accuracy, reaction time). None of the four variables added statistically significant predictive value on participant’s percentage of correct responses (task accuracy; all *R*^2^ < 0.032, all *F*’s < 0.983, all *p*’s > 0.325, with all *β* < 0.194 and all *p’s* > 0.233). For reaction time, on the other hand, it was found that one of the four predictors (i.e., IQ) added statistically significant predictive value in all conditions, except for the condition of full overlap—repetition (*R*^2^ = 0.045, *F*(1,62) = 2.880, *p* = 0.095, with *β* = − 0.212, *p* = 0.095). The results of the regression analyses indicated that IQ explained 9.3% of the variance in the condition no overlap—alteration (*R*^2^ = 0.093, *F*(1,62) = 6.246, *p* = 0.015, with *β* = − 0.305, *p* = 0.015), 6.7% of the variance in the condition full overlap—alteration (*R*^*2*^ = 0.067, *F*(1,62) = 4.395, *p* = 0.040, with *β* = − 0.259, *p* = 0.040), and 7.4% of the variance in the condition no overlap—repetition (*R*^2^ = 0.074, *F*(1,62) = 4.861, *p* = 0.031, with *β* = − 0.074, *p* = 0.031).

### Neurophysiological data

#### Standard event-related potentials (ERP components)

**Figure 3:**
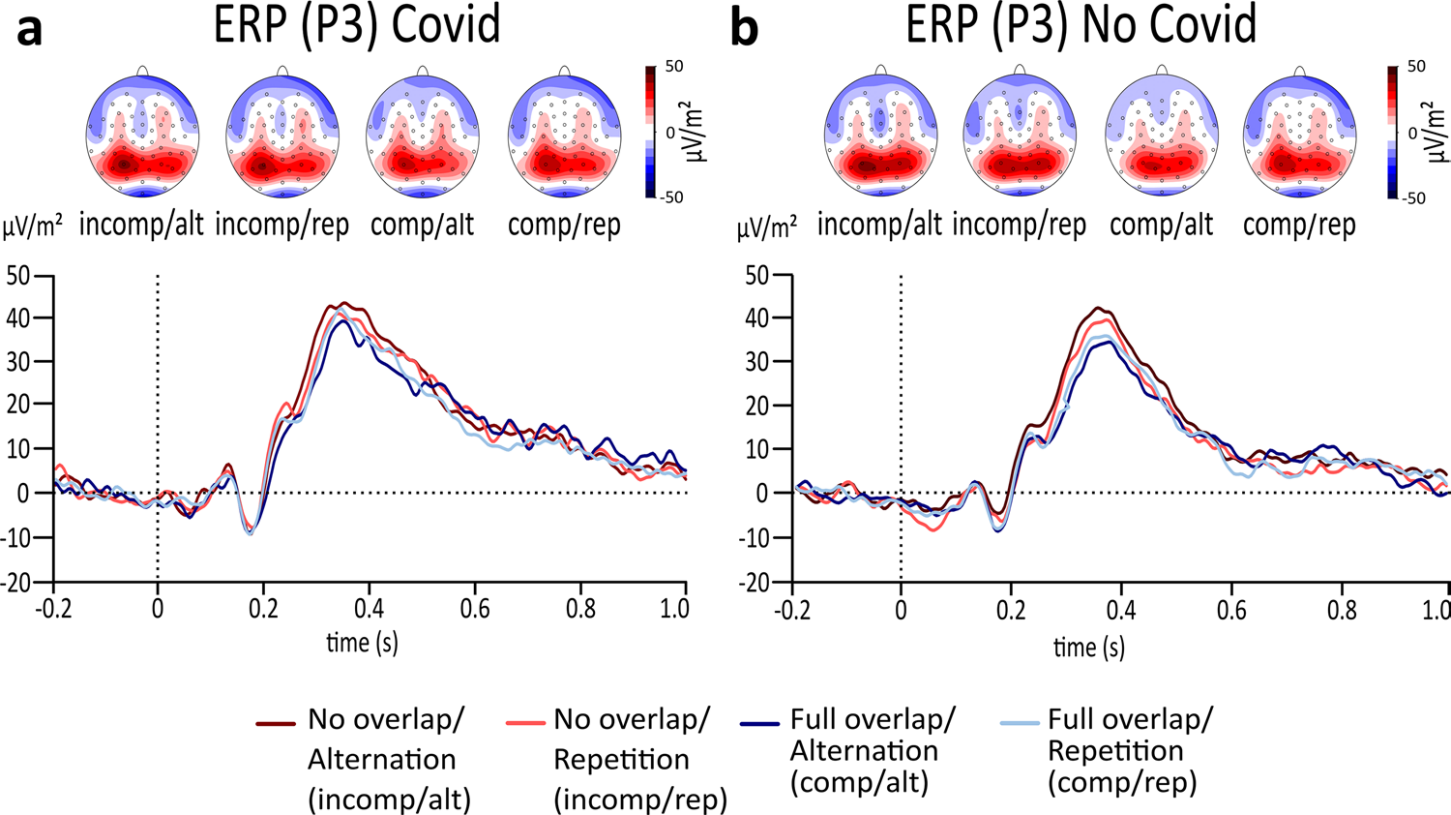
Results of the standard event-related potential (ERP). The plot on the left side (**a**) illustrates standard ERPs at the P3 electrode during the reduced S-R task in the Covid group. The plot on the right side (**b**) illustrates standard ERPs at the P3 electrode during the reduced S-R task in the No Covid group. The alternation condition is illustrated in dark red colour during no overlapping trials and illustrated in dark blue in full overlapping conditions. Repeating trials are either illustrated in light red for no overlapping conditions or illustrated in light blue in fully overlapping conditions. While the x-axis displays the time in seconds (s) based on the onset of the target, the y-axis shows the ERP’s amplitude in μV/m^2^. The scalp topographies illustrate the peak’s potential distribution, showing red colours for positive potentials and blue colours for negative potentials.

The repeated measures ANOVA of the ERP at the P3 electrode revealed a main effect of feature overlap (*F*(1,61) = 16.942, *p* =  < 0.001, *η*_*p*_^2^ = 0.217, *BF*_01_ =  < 0.001) while no main effects of response type or group were found (all *F*’s < 0.944, all *p*’s > 0.335, all *η*_*p*_^2^ < 0.015). Importantly, a significant interaction effect of feature overlap and response type was revealed (*F*(1,61) = 12.186, *p* =  < 0.001, *η*_*p*_^2^ = 0.167, *BF*_01_ = 0.196). The Bayes factor further revealed that there is substantial evidence for this interaction effect. Please refer to Fig. 3 for a detailed illustration of this task effect. Consistent with the behavioural analysis, no significant three-way interaction of response type x feature overlap x group could be found (*F*(1,61) = 0.020, *p* = 0.887, *η*_*p*_^2^ < 0.001, *BF*_01_ = 4.233). Bayesian statistics of this three-way interaction showed that there is substantial evidence for the null hypothesis. Thus, while the analysis of the P3 electrode showed evidence for the established task effect on a neurophysiological level, no differences were found between the two groups, consistent with the behavioral data.

#### RIDE

**Figure 4:**
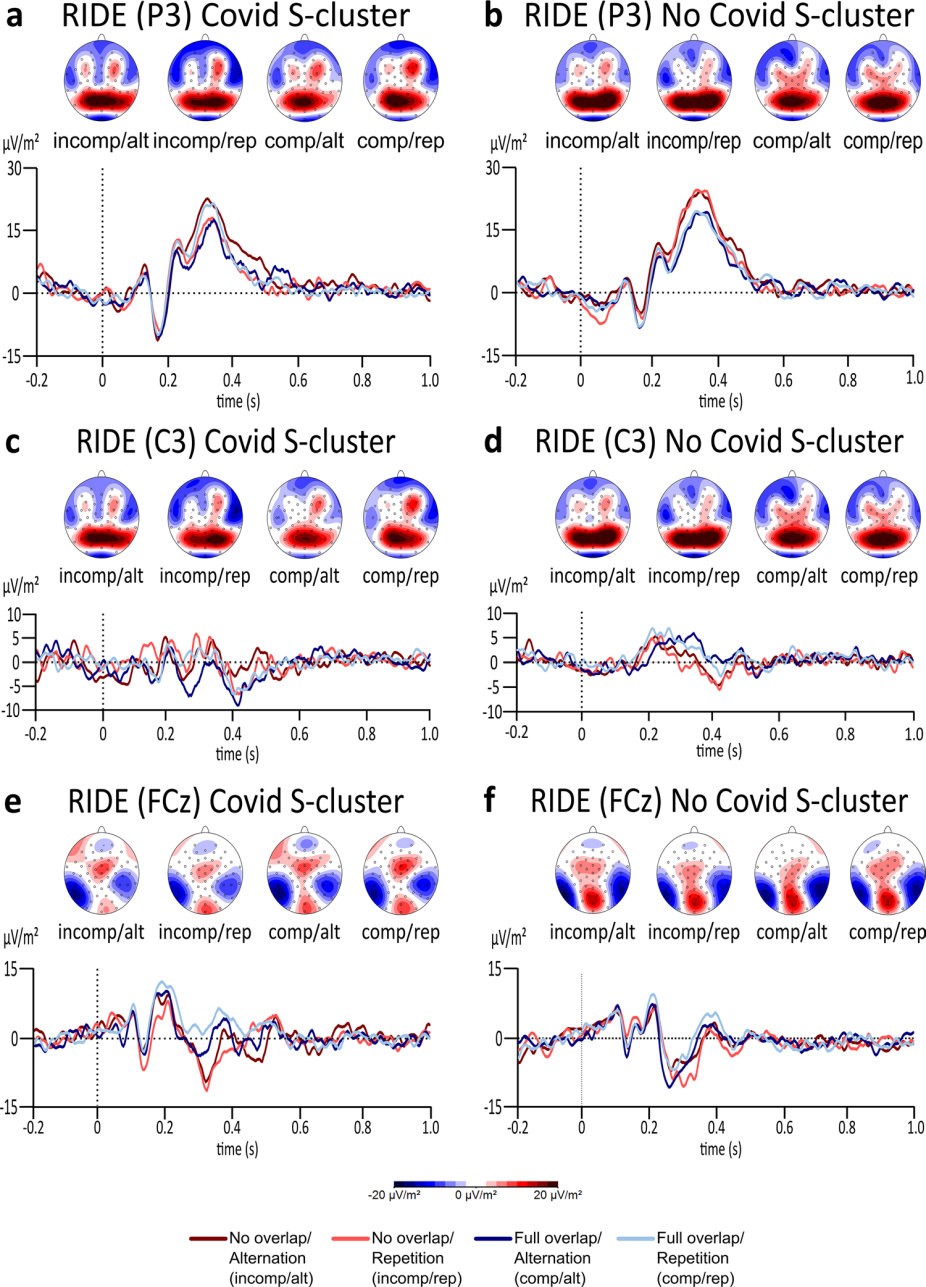
The plots display the RIDE analysis results for the S-cluster at the P3, C3, and FCz electrode for the Covid (**a**, **c**, **e**) as well as for the No Covid (**b**, **d**, **f**) group. The alternation condition is illustrated in dark red colour during no overlapping trials and illustrated in dark blue in full overlapping conditions. Repeating trials are either illustrated in light red for no overlapping conditions or illustrated in light blue in fully overlapping conditions. While the x-axis displays the time in seconds (s) based on the onset of the target, the y-axis shows the ERP’s amplitude in μV/m^2^. The scalp topographies illustrate the peak’s potential distribution, showing red colours for positive potentials and blue colours for negative potentials.

Data for the S-cluster is presented in Fig. 4. While the analysis of the S-cluster at the P3 electrode showed a significant main effect of feature overlap (*F*(1,61) = 14.072, *p* =  < 0.001, *η*_*p*_^2^ = 0.187, *BF*_01_ = 0.026), no significant main effect for response type, group, nor the interaction between response type and feature overlap were significant (all *F*’s < 2.241, all *p*’s > 0.140, all *η*_*p*_^2^ < 0.035). Importantly, no significant interaction effect between response type × feature overlap × group was found either (*F*(1,61) = 0.898, *p* = 0.347, *η*_*p*_^2^ = 0.015, *BF*_01_ = 112.542). The Bayes factor also provides extreme evidence for a lack of group differences at the process of perception–action integration. Moreover, the mixed effects ANOVA at the C3 electrode for the S-cluster did not reveal any significant effects (all *F*’s < 0.861, all *p*’s > 0.357, all *η*_*p*_^2^ < 0.014). Importantly, the Bayes factor for the interaction effect of response type x feature overlap x group at the S-cluster revealed that there is extreme evidence in favour of the null hypothesis for this interaction effect (*BF*_01_ = 1435.984). Similarly, the mixed effects ANOVA at the FCz electrode did not reveal any significant results at the S-cluster (all *F*’s < 2.593, all *p*’s > 0.112, all *η*_*p*_^2^ < 0.041), except a significant main effect for group (*F*(1,61) = 4.078, *p* = 0.048, *η*_*p*_^2^ = 0.063, *BF*_01_ = 8.432). Yet, the Bayes Factor for this main effect provides substantial evidence for the null hypothesis, and thus weakens this result. Again, the Bayes factor for the interaction between response type x feature overlap x group revealed no significant differences at the S-cluster (*BF*_01_ = 687.814). Hence, there is extreme evidence for the null hypothesis.

**Figure 5:**
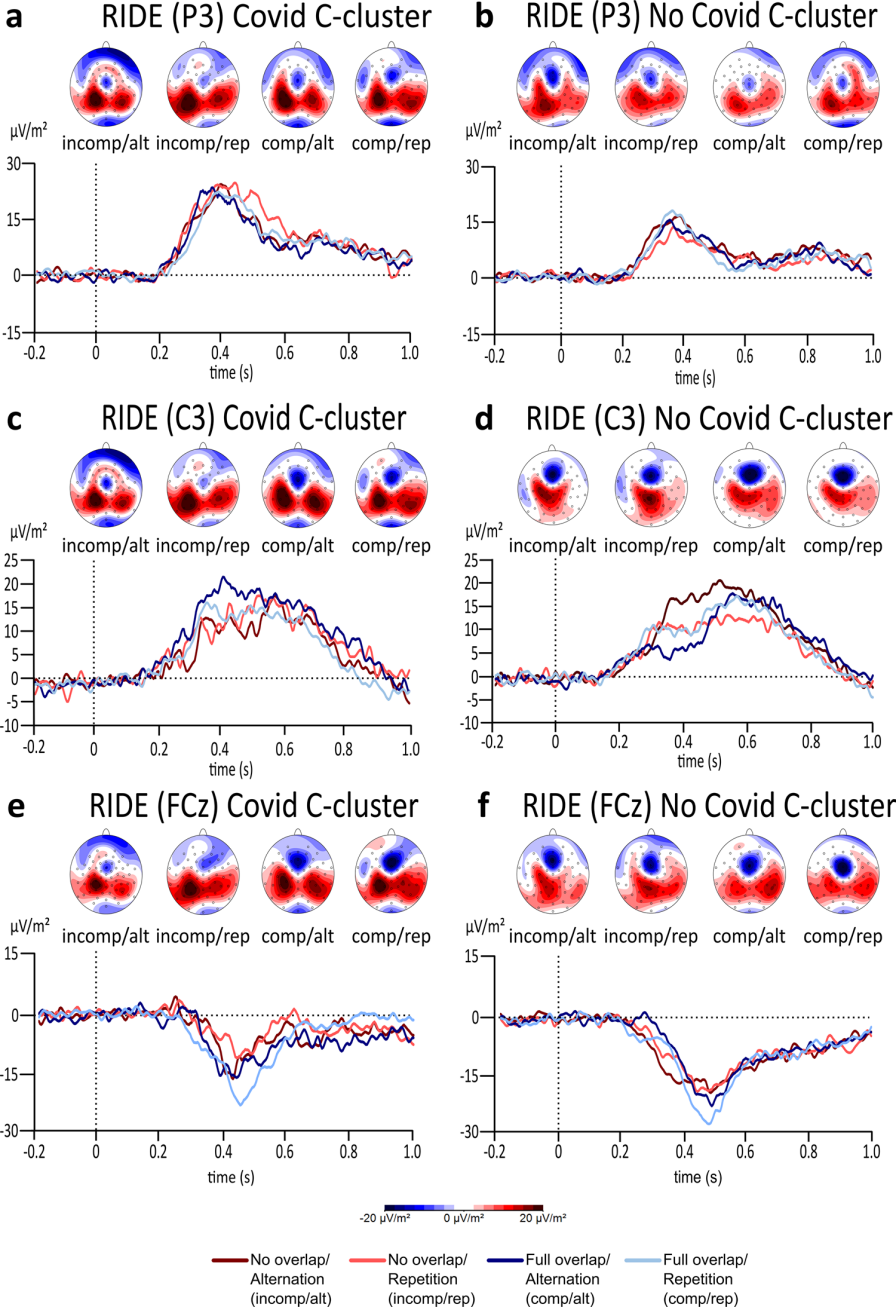
The plots display the RIDE analysis results for the C-cluster at the P3, C3, and FCz electrode for the Covid (**a**, **c**, **e**) as well as for the No Covid (**b**, **d**, **f**) group. The alternation condition is illustrated in dark red colour during no overlapping trials and illustrated in dark blue in full overlapping conditions. Repeating trials are either illustrated in light red for no overlapping conditions or illustrated in light blue in fully overlapping conditions. While the x-axis displays the time in seconds (s) based on the onset of the target, the y-axis shows the ERP’s amplitude in μV/m^2^. The scalp topographies illustrate the peak’s potential distribution, showing red colours for positive potentials and blue colours for negative potentials.

Figure 5 shows the C-cluster. There was a significant main effect of feature overlap (*F*(1,61) = 4.847, *p* = 0.031, *η*_*p*_^2^ = 0.074, *BF*_01_ = 2.992) and group (*F*(1,61) = 4.802, *p* = 0.032, *η*_*p*_^2^ = 0.073, *BF*_01_ = 1.275) but did not reveal any main effect for response type (*F*(1,61) = 0.645, *p* = 0.425, *η*_*p*_^2^ = 0.010, *BF*_01_** = **8.249) at the P3 electrode. More specifically, amplitudes were significantly higher in the full overlap condition (*M* = 19.648, *SE* = 1.633) compared to the no overlap condition (*M* = 16.888, *SE* = 1.633). Moreover, amplitudes were generally higher in the Covid group (*M* = 21.582, *SE* = 2.136) compared to the No Covid group (*M* = 14.953, *SE* = 2.136). Furthermore, no significant two-way (response type x feature overlap; *F*(1,61) = 3.404, *p* = 0.070, *η*_*p*_^2^ = 0.053, *BF*_01_ = 12.583) as well as no three-way interaction (response type x feature overlap x group; *F*(1,61) = 0.292, *p* = 0.591, *η*_*p*_^2^ = 0.05, *BF*_01_ = 117.993) effects were found. Thus, these lack of interaction effects and the high Bayes factors provide strong to extreme evidence for the null hypothesis. Additionally, the mixed effects ANOVA at the C3 electrode for the C-cluster did not reveal any significant effects at all (all *F*’s < 2.860, all *p*’s > 0.096, all *η*_*p*_^*2*^ < 0.045). Importantly, the Bayes factor for the interaction effect of response type x feature overlap x group at the C-cluster revealed that there is extreme evidence for quantifying the null hypothesis (*BF*_01_ = 117.377). Furthermore, the mixed effects ANOVA at the FCz electrode for the C-cluster did not reveal any significant effects at all levels either (all *F*’s < 2.842, all *p*’s > 0.097, *η*_*p*_^2^ < 0.045). The Bayes factor for the interaction effect of response type x feature overlap x group at the C-cluster supports the results in that there are no significant differences between the two groups within the task effect (*BF*_01_ = 568.596).

**Figure 6:**
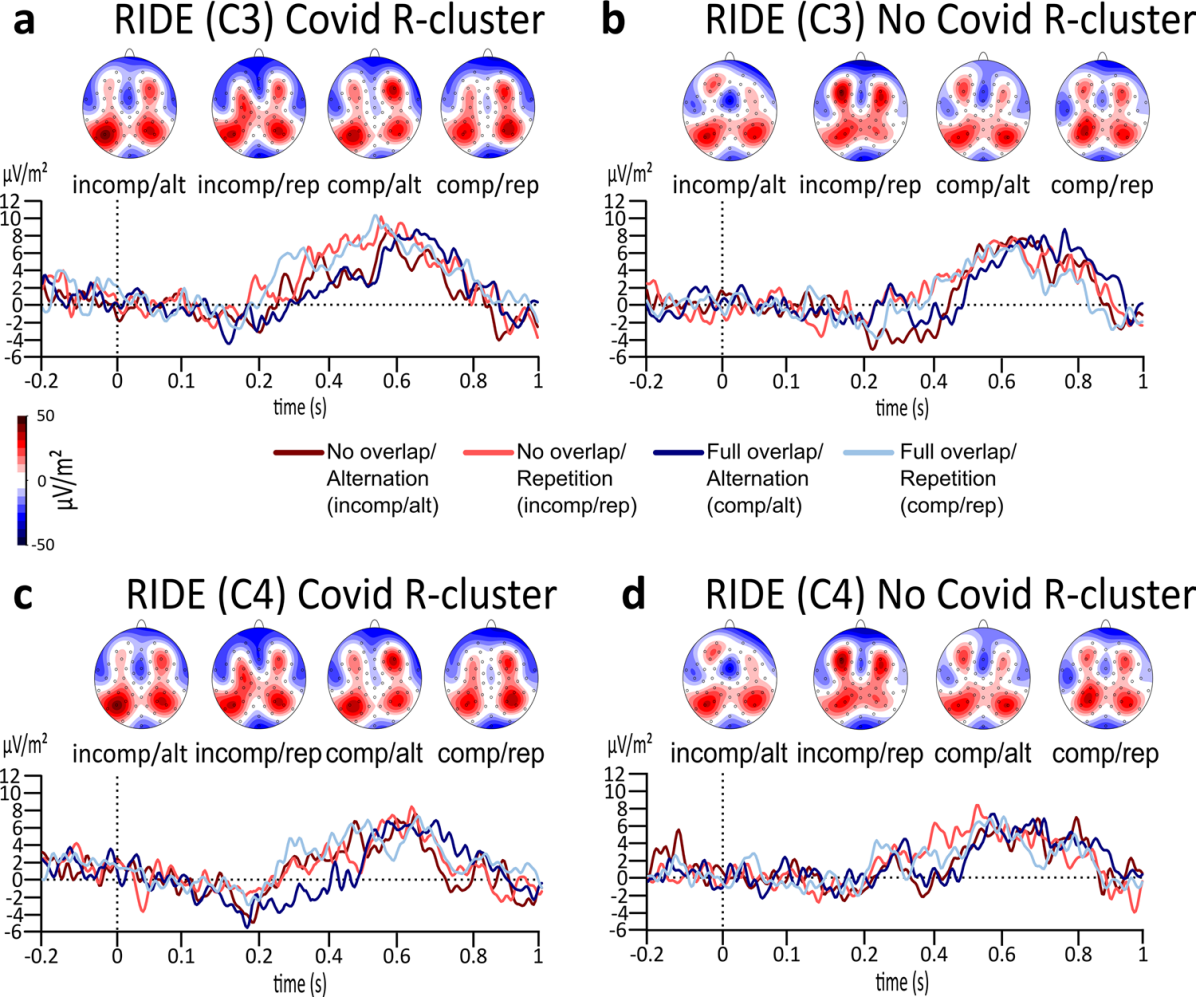
The plots display the RIDE analysis results for the R-cluster at the C3 and C4 electrode for the Covid (**a**, **c**) as well as for the No Covid (**b**, **d**) group. The alternation condition is illustrated in dark red colour during no overlapping trials and illustrated in dark blue in full overlapping conditions. Repeating trials are either illustrated in light red for no overlapping conditions or illustrated in light blue in fully overlapping conditions. While the x-axis displays the time in seconds (s) based on the onset of the target, the y-axis shows the ERP’s amplitude in μV/m^2^. The scalp topographies illustrate the peak’s potential distribution, showing red colours for positive potentials and blue colours for negative potentials.

Figure 6 displays the R-cluster. Besides a significant main effect of response type for the C3 electrode at the R-cluster (*F*(1,61) = 9.238, *p* = 0.003, *η*_*p*_^2^ = 0.132, *BF*_01_ = 0.064), all other main or interaction effects, including the three-way interaction between group × feature overlap × response type (*BF*_01_ = 145.940), have been found to be insignificant (all *F*’s < 0.611, all *p*’s > 0.438, *η*_*p*_^2^ < 0.010). Similarly, results for the C4 electrode at the R-cluster also revealed a significant main effect of response type (*F*(1,61) = 9.469, *p* = 0.003, *η*_*p*_^2^ = 0.134, *BF*_01_ = 0.097),

while no other main or interaction effects, including the three-way interaction between group × response type × feature overlap (*BF*_01_ = 146.025), were found (all *F*’s < 2.826, all *p*’s > 0.098, *η*_*p*_^2^ < 0.044) either.

## Discussion

The current study aimed at examining the impact of a COVID-19 infection on the ability to integrate perception and action in adolescents, a crucial ability to manage daily life abilities^30^. In the present study, no evidence suggesting differences in action-perception integration between adolescents who did and who did not suffer from COVID-19 prior to study participation was found on the behavioural or neurophysiological level. Groups did not differ from each other regarding demographic variables or in scoring pathological questionnaire-reported levels of executive functions and psychiatric symptoms. This study demonstrates an intact functioning of perception–action integration in both examined groups.

In line with earlier research^45,46^, all participants responded more accurately and faster in conditions in which response and stimulus features entirely overlapped or entirely differed from each other but responded significantly slower and less accurately when the same stimulus required a different response. This response pattern was observed independent of COVID-19 serostatus. Notably, the interaction between response type and feature overlap on the behavioural level was also not modulated by factors like current vaccination status or titer value. This, again, substantiates the findings of preserved perception–action integration in adolescence. Along the same lines, group differences in event file binding processes as displayed by a significant interaction effect of response type (alternation, repetition), feature overlap (full or no overlap), and group (Covid, No Covid) were also not found on the neurophysiological level. All participants showed a modulation of the examined EEG component depending on task condition (i.e., a decrease of amplitudes from full feature overlap to no feature overlap conditions when responses had to be repeated and a decrease of amplitudes from no feature overlap to full feature overlap conditions when participants had to alternate their response). This is important, because cognitive functions and underlying neurophysiological processes are subject to considerable intra-individual variability in childhood and adolescence^36^. Especially high intra-individual variability in data can obscure possible differences between groups^38,39^. The current data is unbiased regarding such problems. Multiple lines of evidence (analysis levels in the data) converge to a picture according to which perception–action integration is preserved in adolescents having had COVID-19 infection. How can the observed preserved perception–action integration be explained?

Previous research suggested that cognitive dysfunction following a SARS-CoV-2 infection may result from the ACE-2 receptor allowing the virus to access human cells and areas in the brain/central nervous system that are important for the synthesis of dopamine, consequently leading to an altered dopamine concentration^17,18^. While the ability to integrate perception and action is closely linked to dopaminergic neurotransmission^23,24,27^, several aspects might explain the preserved perception–action integration in younger people as observed in the current study: First, the ACE-2 receptor might be differently expressed in younger people in comparison to adults^15,47–49^. Specifically, the ACE-2 receptor in children or adolescents seems to be lower in number and less functional compared to adults^47,50^. As such, the receptor might have a poorer binding ability to the virus and host cells, making the transport of the virus to the brain and effects there less likely. This may result in a less pronounced modulation of the dopaminergic system and thus reducing the likelihood and/or severity of resulting changes on the cognitive level compared to an adult population. Yet, other factors related to the infection can have an impact on dopamine and cognition, such as levels of stress or psychiatric symptoms, but these factors were controlled for in the current study. Even though the function of the dopaminergic system cannot be directly and non-invasively measured in humans, future studies could aim to apply proxy-measures of dopaminergic functioning (e.g., optical coherence tomography, OCT) in order to assess the association between COVID-19, dopamine, and cognition in more detail^51,52^. Since the dopaminergic system undergoes substantial changes during adolescence^53^, children and adolescents might have a better potential to recover from possible abnormalities in the dopaminergic system induced by a COVID-19 infection or to compensate them. Similarly, the process of event file binding in children and adolescents is also still developing and becomes more efficient as a function of age^33,34^. All of these aspects provide possible explanations for preserved perception–action integration in adolescents as observed in the current study.

There are limitations of the study. Only limited information could be collected about the participant’s course of infection and, in line with literature on SARS-CoV-2 in children and adolescents, most infections go by unnoticed or occurred without or with just mild symptoms^54^. Further, the time span passing between the infection and our EEG appointment was highly variable between participants leading to a potentially distorted view on event file coding following a COVID-19 infection since it is known by now that most symptoms are likely to recover with time^55^. To assess the influence of the time span passed since the infection, future studies should examine the impact of COVID-19 on event file coding in a longitudinal design. Finally, although action-perception integration represents an important cognitive function, cognition generally entails many different aspects with binding being just one of many components of cognitive functioning and cognitive control. Consequently, there is a great need for further studies in other cognitive domains.

In summary, the study provides novel insights into the cognitive control mechanism of action-perception integration in adolescents following a SARS-CoV-2 infection by being one of the first to investigate the underlying neurophysiological mechanisms next to behavioural data in a central cognitive function—perception–action integration. This finding suggests that while cognitive impairments can occur following a COVID-19 infection, action-perception integration as one of the basic building blocks of cognition seems to be largely unaffected in adolescents with a rather mild or asymptomatic course of the disease.

## Materials and methods

### Participants

A total of N = 63 participants between 11 and 19 years of age were recruited for our present study (24 male; M_age_ ± SE = 15.5 ± 0.404 years). This sample size was arrived at based on other study designs investigating the S-R paradigm and achieving good reliability^27^. Prior to the analysis, N = 4 participants were excluded since those were detected as extreme outliers from the group (number of correct trials < 21 for at least one condition). Of the remaining N = 63 participants, N = 34 participants had been infected with COVID-19 prior to the study. These participants are subsequently referred to as the “Covid group”. All other participants had not been infected before study participation (referred to as the “No Covid group”). The time span between Covid-19 infection and study participation could not be controlled for in the current study, because the data were collected based on a larger school-based study, in which blood was drawn from participants numerous times in order to measure antibodies. Hence, no definite statements can be made about the duration of onset of the Covid group. The time span is only known in eleven out of 34 participants, namely on average 6.64 months (*M* = 6.64, *SE* = 0.82). The Covid group had a significantly higher mean titre value (Au/ml; *M* = 228.04, *SE* = 28.73) than the No Covid group (*M* = 60.59, *SE* = 25.71, *t*(61) = -4.28, *p* =  < 0.001). Furthermore, people in the Covid group have significantly more often been vaccinated once before study participation (21 out of 34) than people in the No Covid group (7 out of 29; *χ*^2^(1) = 8.974, *p* = 0.003). The mean IQ in both groups was in the average range, namely 104.82 (± 1.920) for the Covid group and 102.24 (± 2.688) for the No Covid group (*t*(61) = − 0.798, *p* = 0.428). To screen for the presence of psychiatric symptoms and other factors potentially influencing task performance, participants and one of their parents reported on the subjective quality of life, the level of executive functioning, and psychiatric symptoms using three online questionnaires. The questionnaires used were the KINDL (3–17 years of age^56^), the Behaviour Rating Inventory of Executive Functioning (BRIEF/11–16 years of age^57^), and the Youth Self-Report (YSR/11–18 years of age)/Child Behaviour Checklist (CBCL^58^). The two groups differed significantly from each other on the symptom scale ‘thought problems’ of the YSR (*t*(52) = 2.73, *p* = 0.009**)** and the symptom scale ‘inhibit’ of the parent’s report of the BRIEF (*t*(53) = − 2.05, *p* = 0.04). However, scores of both groups were outside of the clinically relevant range, therefore indicating no relevant level of psychiatric symptoms or executive dysfunctions in either of the groups. Please refer to Table 1 for a more complete overview of the descriptive information of the sample. Due to the non-compliance of ten of the 63 participants, complete questionnaire data was not available for all participants. The ethics committee of the Faculty of Medicine of the TU Dresden approved the study (SR-EK-8012020) which was conducted according to all relevant guidelines. Participants or their legal guardians signed an informed consent prior to the study and did not receive any reimbursement for participation.

**Table 1:** Demographics of the Sample (N_Covid_ = 34, N_No Covid_ = 29).

Demographic variables	Covid Group (m ± SE)	No Covid Group (m ± SE)	*t*(df) = ,* p* =
Gender (Valid *N*)			*χ*^2^(1) = 3.57,* p* = 0.059
Male	10	14	
Female	24	15	
Age	15.94 ± 0.34	15.45 ± 0.32	*t*(61) = − 1.04, *p* = 0.301
IQ	104.82 (± 2.69)	102.24 (± 1.92)	*t*(61) = − 0.80,* p* = 0.428
Vaccinated	21	7	*χ*^2^(1) = 8.97, *p* = 0.003**
Titer (Au/ml)	228.04 ± 28.73	60.59 ± 25.71	*t*(61) = − 4.28,* p* = < 0.001***
Self-report: YSR/11–18 years of age			
Withdrawn/depressed	58.33 ± 1.09	57.08 ± 1.64	*t*(52) = 0.66, *p* = 0.515
Somatic complaints	59.93 ± 1.61	57.25 ± 1.82	*t*(52) = 1.11, p = 0.274
Anxious-depressed	56.40 ± 1.96	56.67 ± 1.92	*t(*52) = − 0.10, p = 0.924
Social problems	56.03 ± 1.01	53.79 ± 1.51	*t*(52) = 1.27, *p* = 0.209
Thought problems	65.07 ± 1.24	59.63 ± 1.61	*t*(52) = 2.73, *p* = 0.009**
Attention problems	57.90 ± 1.44	55.33 ± 1.45	*t*(52) = 1.24, *p* = 0.221
Rule-breaking behaviour	55.73 ± 0.91	54.79 ± 0.98	*t*(52) = 0.70, *p* = 0.485
Aggressive behaviour	54.60 ± 0.98	52.96 ± 0.87	*t*(52) = 1.22, *p* = 0.216
Self-report: BRIEF/11–16 years of age			
Inhibit	46.06 ± 1.88	44.00 ± 0.93	*t*(58) = 0.96, *p* = 0.330
Shift	49.10 ± 1.72	47.45 ± 1.60	*t*(58) = 0.70, *p* = 0.480
Emotional control	48.90 ± 1.82	48.21 ± 1.61	*t*(58) = 0.29, *p* = 0.777
Monitor	47.26 ± 1.75	45.38 ± 1.36	*t*(58) = 0.84, *p* = 0.404
Working memory	49.87 ± 2.24	48.90 ± 2.23	*t*(58) = 0.32, *p* = 0.754
Plan/organize	49.94 ± 1.86	49.79 ± 1.87	*t*(58) = 0.05, *p* = 0.957
Organization of materials	51.03 ± 2.37	51.76 ± 1.47	*t*(58) = − 0.26, *p* = 0.799
Task completion	46.35 ± 1.68	50.24 ± 2.34	*t*(58) = − 1.36, *p* = 0.178

Demographic variables	Covid (M ± SE)	No Covid (M ± SE)	*t*(df) = *, p*
Self-report: KINDL/3–17 years of age			
Physical well-being	62.30 ± 2.85	71.34 ± 2.85	*t*(58) = − 1.99, *p* = 0.052
Emotional well-being	72.18 ± 3.04	77.37 ± 3.24	*t*(58) = − 1.17, *p* = 0.247
Self-esteem	66.13 ± 3.43	59.48 ± 3.94	*t*(58) = 1.28, *p* = 0.207
family	81.25 ± 2.93	84.48 ± 3.08	*t*(58) = − 0.76, *p* = 0.449
Friends	69.56 ± 3.44	76.72 ± 2.84	*t*(58) = − 1.60, *p* = 0.116
School	64.11 ± 2.81	61.42 ± 3.59	*t*(58) = 0.59, *p* = 0.555
Parent’s report: CBCL/11–18 years of Age			
Withdrawn/depressed	53.63 ± 1.29	53.31 ± 0.97	*t*(51) = − 0.20, *p* = 0.843
Somatic complaints	56.61 ± 1.48	54.38 ± 1.39	*t*(52) = − 1.09, *p* = 0.281
Anxious/depressed	53.11 ± 0.83	53.46 ± 1.27	*t*(52) = 0.237, *p* = 0.813
Social problems	53.70 ± 1.11	51.38 ± 0.91	*t*(51) = − 1.62, *p* = 0.113
Thought problems	55.25 ± 1.20	52.69 ± 1.05	t(52) = − 1.60, *p* = 0.116
Attention problems	53.21 ± 1.32	51.00 ± 0.57	*t*(52) = − 1.55, *p* = 0.131
Rule-breaking behaviour	52.07 ± 0.90	50.73 ± 0.73	*t*(52) = − 1.15, *p* = 0.257
Aggressive behaviour	51.89 ± 0.66	51.35 ± 0.76	*t*(52) = − 0.55, *p* = 0.588
Parent ‘s report: BRIEF/11–16 years of age			
Inhibit	45.31 ± 1.08	42.69 ± 0.60	*t*(53) = − 2.05, *p* = 0.04*
Shift	44.14 ± 1.39	43.27 ± 1.17	*t*(53) = − 0.47, *p* = 0.638
Emotional control	59.83 ± 16.67	43.50 ± 1.18	*t*(53) = − 0.93, *p* = 0.359
Initiate	44.76 ± 1.64	44.81 ± 1.85	*t*(53) = 0.02, *p* = 0.984
Working memory	42.62 ± 1.47	41.81 ± 1.33	*t*(53) = − 0.41, *p* = 0.685
Plan/organize	41.86 ± 1.40	43.19 ± 1.77	*t*(53) = 0.60, *p* = 0.553
Organization of material	47.24 ± 1.53	44.96 ± 1.89	*t*(53) = − 0.95, *p* = 0.348
Monitor	43.00 ± 1.78	41.19 ± 1.56	*t*(53) = − 0.755, *p* = 0.454

Demographic variables	Covid (M ± SE)	No Covid (M ± SE)	*t*(df) = *, p*
Parent’s report: KINDL/3–17 years of age			
Physical well-being	85.13 ± 3.05	83.65 ± 2.48	*t*(53) = − 0.37, *p* = 0.712
Emotional well-being	84.06 ± 2.09	85.58 ± 1.83	*t*(53) = 0.54, *p* = 0.590
Self-esteem	74.57 ± 2.58	72.36 ± 2.46	*t*(53) = − 0.62, *p* = 0.539
Family	83.62 ± 2.33	85.10 ± 2.05	*t*(53) = 0.47, *p* = 0.641
Friends	79.96 ± 2.85	78.13 ± 2.89	*t*(53) = − 0.45, *p* = 0.654
School	78.45 ± 2.90	75.96 ± 2.94	*t*(53) = − 0.60, *p* = 0.550

0.01 ≤ *p* < 0.05: *; 0.001 ≤ *p* < 0.01: **; *p* < 0.001: ***

### Task

**Figure 7:**
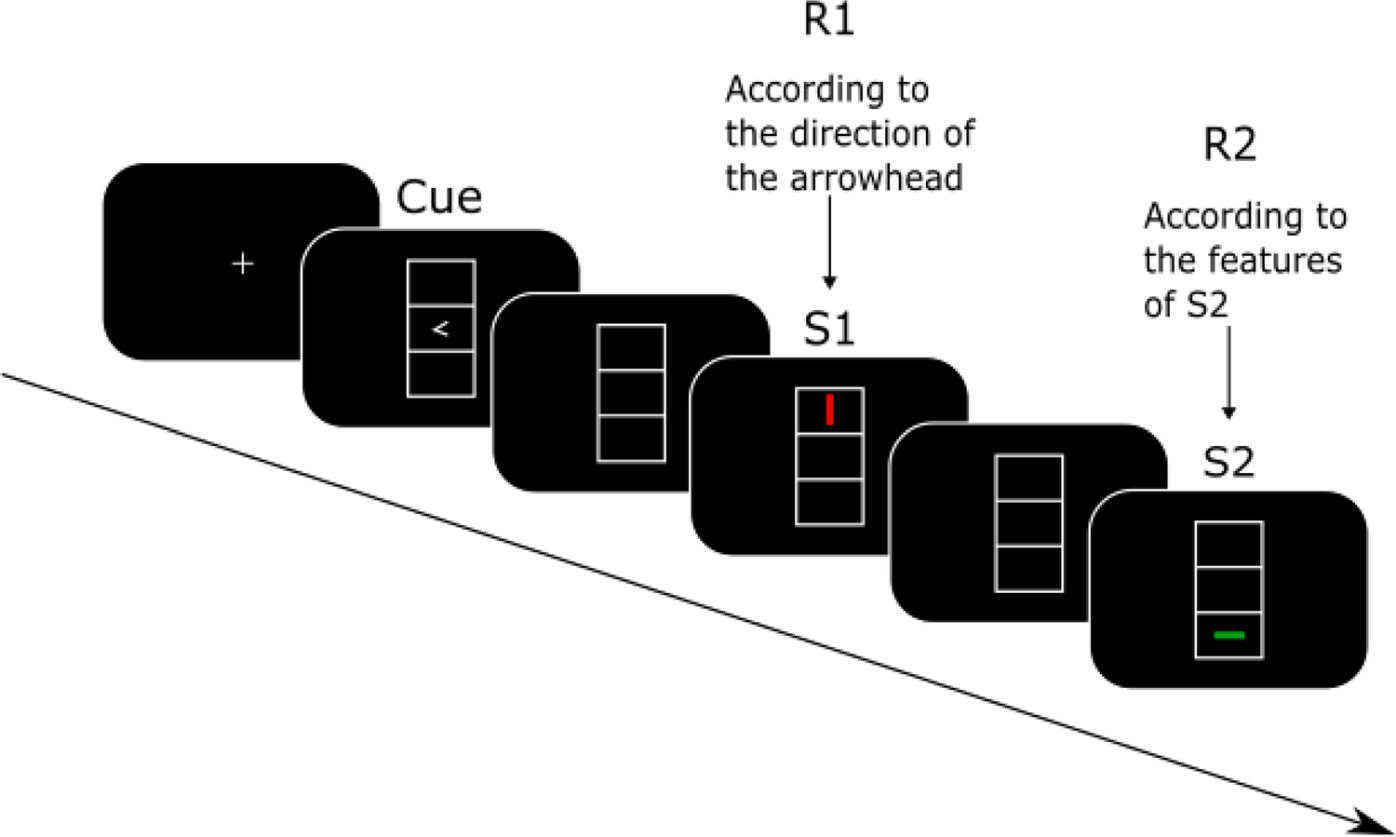
The sequence of stimuli and response cues in the S-R-R task. Please refer to the Method section for further information regarding the timing, etc. Adopted from Eggert et al.^25^.

An adapted version of the stimulus-response (S-R) task was used in the present study to assess event file binding in participants who had a COVID-19 infection prior to the study compared to those who did not. The established standard paradigm, originally developed by Colzato and colleagues^35^, was reduced from four to two feature overlap conditions only (S-R-R) since previous research showed that the „zero feature “ and „full feature “ overlap conditions in differing repetition and alternation response trials are the most important for reliably analysing the process of event file coding^27,43,44^. Participants were seated in front of a 24-inch computer screen that was placed at approximately 60 cm distance to the participants. After performing a number of practice trials, the actual task consisted of 192 trials, which were further divided into six blocks of 32 trials. The general sequence of the task was the following: after a response cue appeared on the screen, two stimuli followed one after the other (see Fig. 7). The response cue and the stimuli were all presented in one of the three vertically aligned boxes (each 2.4 × 0.9 cm). More precisely, after a fixation cross was presented on a blank screen between the trials for 1500–2000 ms, the response cue (an arrowhead that either pointed to the left or to the right) was displayed for 1500 ms in the middle box. Consecutively, the first stimulus (S1) appeared for 500 ms followed by the second stimulus (S2) which was shown for 2000 ms or until the participants had given their response. In between the response cue and S1, an empty screen was presented for 1000 ms as well as for 2000 ms between S1 and S2. The two stimuli either appeared in the top or bottom box and further differed in colour (red, green) and orientation (horizontal, vertical). Thus, the stimuli of the task differed in three different features (location, colour, orientation). Two responses were required per trial. Participants were instructed to withhold their immediate response to the response cue and to only press a button (R1) according to the direction of the arrowhead at the appearance of stimulus S1. Incorrect responses lead to a repetition of the trials (up to three times). Importantly, previous research showed that the emerging relationship between S1 and R1 elicits an automatic event file binding between the two^31^. Subsequently, at the time point of the appearance of S2, participants had to respond to the features of the stimulus itself (R2). Participants were required to press the left control key button for a horizontally displayed bar and the right control key button for a vertically displayed bar. Since the used reduced task version left out the „partial feature overlap “ conditions in order to reduce the length of the experiment, S1 and S2 differed either fully from each other („no overlap “ condition, i.e., S1 and S2 do not share any features) or were equivalent to each other („full overlap “ condition, i.e., S1 and S2 share all features). Hence, participants either had to repeat their responses by pressing the same key twice or had to alternate their responses by pressing a different key during one task trial. Generally, it has been shown that people respond slower and less accurately in situations in which the same stimulus requires a different response (either response or stimulus features differ). On the other hand, people respond faster and more accurately when they are confronted with complete repetitions (both response and stimulus features entirely overlap) or with complete alternations (both response and stimulus features entirely differ from each other). In line with the Theory of Event Coding (TEC), this results from a difficulty of a parallel retrieval and unbinding of previously established event files^45,46^.

### EEG recording and analysis

We made use of 60 Ag/AgCl electrodes to record the EEG including the Fpz as the reference electrode, the ground electrode being kept at θ = 58, φ = 78, and electrode impedance smaller than 5 kΩ. Using BrainVision Analyzer 2.1, the EEG data was pre-processed and analysed. While pre-processing the data offline, the sampling rate was changed to 256 Hz and a band-pass filter of 0.5–40 Hz was applied. While technical artefacts were removed during manual inspection of the data, artefacts that occurred periodically (horizontal and vertical eye movements, pulse artefacts) were corrected during an independent component analysis (ICA, infomax algorithm). Afterwards, the data were segmented to the stimulus onset of S2, namely 2000 ms before and 2000 ms after the presentation of the stimulus. Trials were only included in the segmentation when both, the R1 and the R2 were correct and when the R1 within 500 ms after the appearance of S1. The R2 response time limit was 2000 ms after the presentation of S2. In total, four different segments were created based on the response type (alternation vs repetition) and feature overlap (no vs full overlap) levels. Following this, an automated artefact rejection procedure was applied with the following rejection criteria: intervals with amplitudes more than ± 150 µV, as well as with activity below 0.5 µV for 100 ms were discarded and marked as bad interval 200 ms prior and 200 ms after the event. Subsequently, a current source density (CSD) transformation was applied (4 splines and 10 polynomials). The CSD transformation, which works as a spatial filter, makes it easier to identify relevant electrode sites^59^. After a baseline correction (− 200–0 ms prior to the onset of S2), averages were calculated individually for each condition and participant. Based on that, grand averages were calculated separately for the conditions. All electrodes and time windows were chosen according to standard procedures. By visually inspecting the scalp topography, electrode P3 was chosen to be analysed in the time window between 320 and 380 ms after S2 onset. It has been shown that relevant time windows and electrodes can be different between younger people and adults^33^, hence making it difficult to rely on information from studies that included adults only^43,44^.

### Residue iteration decomposition

Residue iteration decomposition (RIDE) was used for further processing of the data^38,39^. By that, it was possible to differentiate between different coding levels in the EEG signal that are critical to distinguish during event file processing and which are usually intertwined within event-related potentials (ERPs). At the same time, inter- and intra-individual variability can be accounted for which is especially important in studies including younger participants^36^. RIDE was applied in Matlab (Mathworks, Inc., Natick, MA) based on existing procedures^27,37,39^ and using the RIDE toolbox (see http://cns.hkbu.edu.hk/RIDE.htm for a manual). RIDE decomposition is applied on single-trial ERPs and separately for each electrode based on latency variability of various components. The S (“stimulus”) and R (“response”) clusters were obtained by using latency information of stimulus and response onset. Latency information for the C (“central”) cluster was estimated and iteratively improved in each trial. Time windows of the different clusters were chosen according to a study also studying perception–action integration in a younger sample^33^ and were as follows: 200 ms before the onset of S2 until 700 ms after the onset of S2 for the S-cluster, 300 ms prior and after the onset of R2 for the R-cluster and 150 ms until 800 ms after the presentation of S2 for the C-cluster. Please refer to Ouyang et al^38,39^ for detailed information on RIDE.

To quantify the RIDE-decomposed data, we first chose electrode P3 which had been identified during the standard ERPs for data analysis. Furthermore, electrodes C3 and FCz were chosen based on plots of scalp topography and the related time windows were selected based on visual inspection. This was done, because amplitude latencies and electrodes possibly differ between samples of adults and children^33^ and because the topography can change after applying the RIDE procedure^36^. This was done for the two groups and each condition. Hence, the time intervals for the S-cluster at the P3 electrode ranged from 295 to 370 ms in the Covid group and from 320 to 385 ms in the No Covid group. For the C-cluster at the P3 electrode, the time interval between 355 and 425 ms was selected for the Covid group and 320–410 ms in the No Covid group. At the C3 electrode, the time intervals ranged from 380 to 440 ms in the Covid group and 485 to 545 ms in the No Covid group within the C-cluster, as well as 280 to 340 ms in the Covid group and 320 to 380 ms in the No Covid group within the S-cluster. Finally, at the FCz electrode, the time intervals ranged from 420 to 480 ms in the Covid and No Covid group within the C-cluster and from 160 to 220 ms for the Covid group and 180–240 ms in the No Covid group within the S-cluster. Moreover, electrodes C3 and C4 have been selected for analysis in the R-cluster to examine possible motor cortex processes. For that, time intervals were chosen based on the mean reaction times for each condition within each group for the same time range as chosen before (60 ms; see Fig. 2).

### Design and statistical analysis

JASP 0.16.2.0 (JASP team, 2022) and SPSS (version 28) were used to conduct the statistical analysis of the behavioural and EEG data. The group membership of the participants was not known during the pre-processing stage of the data (blinded analysis) in order to avoid potential biases. First, mean reaction time and mean accuracy (percentage of correct responses) were calculated for the behavioural analysis. A 2 × 2 × 2 mixed effects analysis of variance (ANOVA) was performed for task accuracy and reaction time as well as a repeated measures ANOVA was used to analyse the neurophysiological data at hand. For both analyses, the „group “factor (Covid vs No Covid) served as the between-subject factor, whereas „response type “(repetition vs alternation) and „feature overlap “(full overlap vs no overlap) were the within-subject factors. A significant interaction effect of the factors “response type”, “feature overlap” and “group” would reflect existing differences in the event file binding processes between the two groups. 0.05 (α = . 05) was the chosen alpha threshold for the significance level. Importantly, we corrected all post-hoc comparisons with Bonferroni-correction. In case of significant or non-significant interaction effects between group x response type x feature overlap, Bayesian statistics were also used to estimate the strength of the significant or null findings (i.e., that there are or that there are no differences of event file binding between groups^60,61^). The Bayes Factor for H_01_ was calculated using the Bayesian Repeated Measures ANOVA in JASP and the classification scheme of the Bayes Factor, as proposed in Wagenmakers and colleagues^62^: > 100 = extreme evidence for the null hypothesis (H_0_), values between 30 and 100 = very strong evidence for H_0_, ten to 30 = strong evidence for H_0_, values between three to ten = substantial evidence and values between one and three provide anecdotal evidence for H_0_. Conversely, values between 1/3 and 1 provide anecdotal evidence for the alternative hypothesis (H_1_), 1/10–1/3 provide substantial evidence for H_1_, 1/30–1/10 provide strong evidence for H_1_, values between 1/100 and 1/30 provide very strong evidence for H_1_ and values smaller than 1/100 provide extreme evidence for H_1_. Normal distribution of the data was checked by the Shapiro–Wilk test. Furthermore, a backward stepwise linear regression was performed in order to explore the influence of potential predictors (vaccination status, serostatus, titre value, IQ) on participant’s task performance.

## Data Availability

The corresponding author will provide the generated datasets that have been analysed during the current investigation upon reasonably request.

## Abbreviations

ACE-2: Angiotensin-converting enzyme 2
COVID-19: Coronavirus disease 2019
CSD: Current source density
ERP: Event-related potential
ICA: Independent component analysis
RIDE: Residue iteration decomposition
SARS-CoV-2: Severe acute respiratory syndrome coronavirus 2
S-R: Stimulus-response
S-R-R: Stimulus-response-reduced
TEC: Theory of event coding
